# Pancoast-Tobias Syndrome: A Unique Presentation of Lung Cancer

**DOI:** 10.7759/cureus.13112

**Published:** 2021-02-03

**Authors:** Mian Munir, Saad Bin Jamil, Sameerah Rehmani, Carolina Borz-Baba

**Affiliations:** 1 Internal Medicine, Saint Mary’s Hospital's Internal Medicine Residency Program, Waterbury, USA; 2 Hospitalist Medicine/Internal Medicine, Saint Mary's Hospital, Waterbury, USA; 3 Internal Medicine, Sentara Obici Hospital, Suffolk, USA; 4 Internal Medicine, Saint Mary's Hospital, Waterbury, USA

**Keywords:** pancoast tumor, bronchogenic adenocarcinoma, horner’s syndrome

## Abstract

A 65-year-old man with 50 pack-year smoking history presented to the emergency department for evaluation of upper back and right shoulder pain secondary to a fall. Physical examination was notable for anisocoria with a constricted left pupil (miosis), mild ptosis of the left eyelid, and bilateral shoulder pain, right more than left, with both passive and active movements. Chest computed tomography identified a soft tissue mass at the left lung apex with extension into the pleural surface, associated with destructive osseous changes of the right scapula, adjacent ribs, and thoracic vertebral bodies. Imaging of the brain revealed multiple masses suspicious of metastatic brain lesions. Biopsy of the right supraclavicular lymph node revealed lung tissue adenocarcinoma and negative Kirsten rat sarcoma viral oncogene homolog (K-Ras), epidermal growth factor receptor (EGFR), B-raf proto-oncogene (BRAF), C-ros oncogene 1 (ROS1), and anaplastic lymphoma kinase (ALK) rearrangement.

Recognizing Pancoast syndrome in patients with significant smoking history, anisocoria, and shoulder pain is crucial for identifying the underlying etiology and expediting the treatment.

## Introduction

Pancoast tumor is a neoplasm located at the apex of the lung with characteristic involvement of apical chest wall and thoracic inlet structures, which accounts for less than 5% of bronchogenic carcinoma. The clinical presentation of the Pancoast tumor is distinctive. Prompt diagnosis requires an understanding of the anatomy of the affected region. Pancoast tumors are located at the apex of the lungs. The thoracic inlet present at this site is a narrow compartment and has several important neurovascular structures passing through it. The involvement of these structures leads to a constellation of symptoms based on the structure affected.

The thoracic inlet is divided into three components, namely anterior, middle, and posterior. The anterior compartment involvement affects the subclavian and jugular veins which may result in pain radiating to the upper chest and venous thrombosis of adjacent veins. The middle compartment has trunks of the brachial plexus and phrenic nerve, which can lead to pain and paresthesia of the shoulder and upper limb as in our patient’s case, as well as paralysis of the diaphragm. The presence of a subclavian artery in the vicinity of the tumor may lead to arterial thrombosis. The posterior compartment contains the stellate ganglion, the sympathetic chain, muscular components, and tumor spread to this area results in pain of the axillary region and upper arm and classically Horner’s syndrome. The majority of patients with Pancoast tumors present with one or more of these symptoms. Involvement of the upper ribs, brachial plexus, and vertebral bodies results in shoulder pain.

Most Pancoast tumors are non-small cell lung cancer (NSCLC). The most common is the squamous cell in origin (52%), followed by adenocarcinomas (23%), and large cell carcinomas (20%). Only about 5% of Pancoast tumors are of small cell origin [1]. The prognosis has been historically poor; however, with the advent of induction chemo-radiotherapy, there has been an improvement in survival rates.

We report a case of a patient with bronchogenic adenocarcinoma who presented with anisocoria as a predominant clinical manifestation of Pancoast tumor.

## Case presentation

A 65-year-old man presented to the emergency department for evaluation of fall with back and right shoulder pain. On further questioning, he endorsed dysphagia and about 50-pound unintentional weight loss in the last month. He was an active smoker who had smoked a pack per day for about 50 years. In the ED, he was alert but only oriented to time. He complained of severe pain in his back and right shoulder. His friends had noticed his declining health in the last month and noticed slurring in his speech.

Physical examination was pertinent for cachexia (BMI-16) with temporal wasting. He had slurred speech with a hoarse voice, which made it difficult to understand him. He had unequal pupils with a miotic left pupil. The anisocoria was appreciated more in the darkness, as the left pupil was unable to dilate. Mild ptosis of the left eyelid was noted. He also had a significantly noticeable enlarged left supraclavicular/Virchow’s node, bilateral axillary lymphadenopathy, and digital clubbing. The lung auscultation was significant for diffuse bilateral rhonchi and wheezing predominantly in the upper lung fields. The musculoskeletal examination demonstrated a limited range of motion with both active and passive movements of the shoulders, weakness of right shoulder abduction above the horizontal plane secondary to severe pain, and a three of five strength of the left upper extremity.

**Figure 1:**
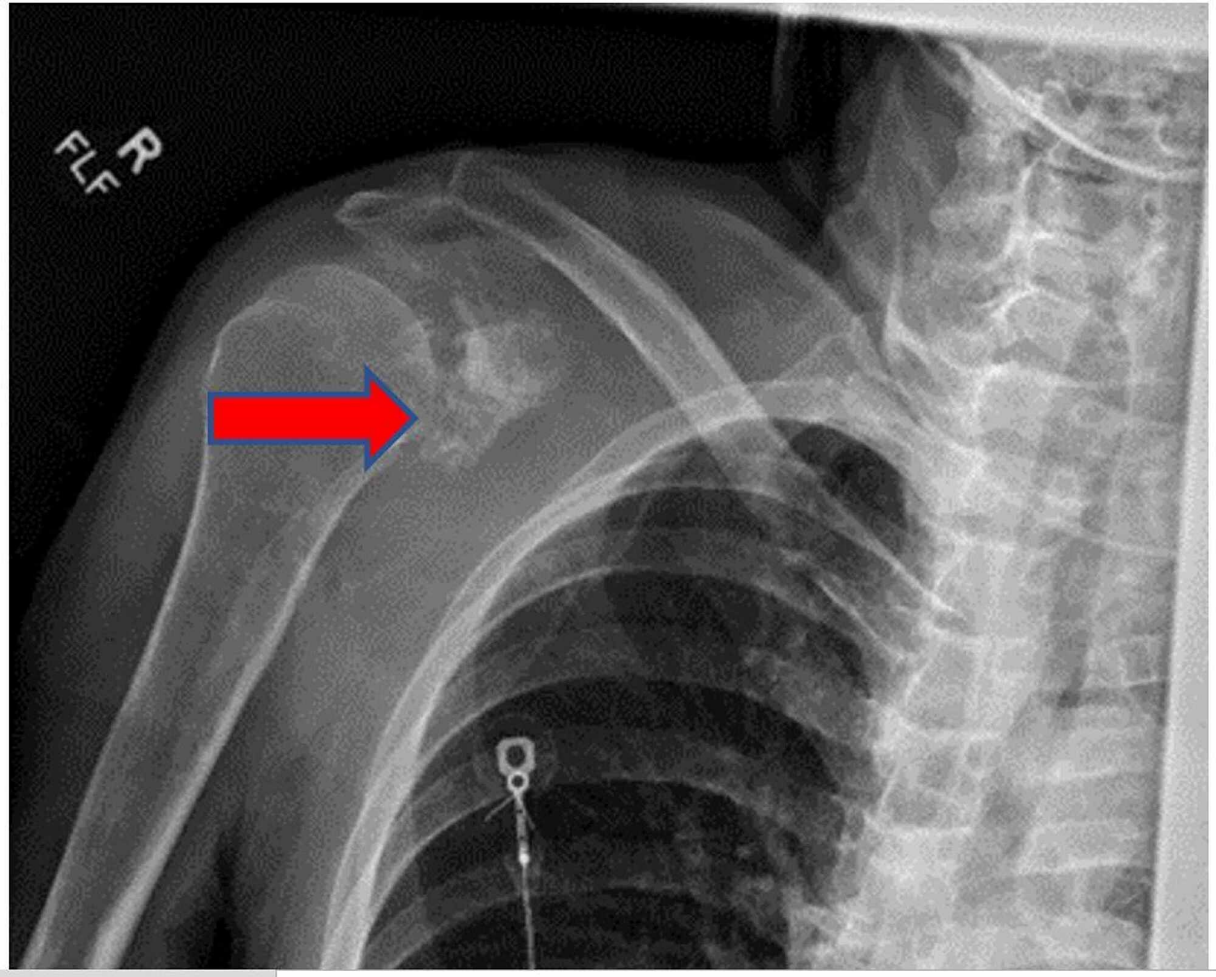
Right shoulder plain x-ray revealing bony destruction of scapula, (red arrow).

**Figure 2:**
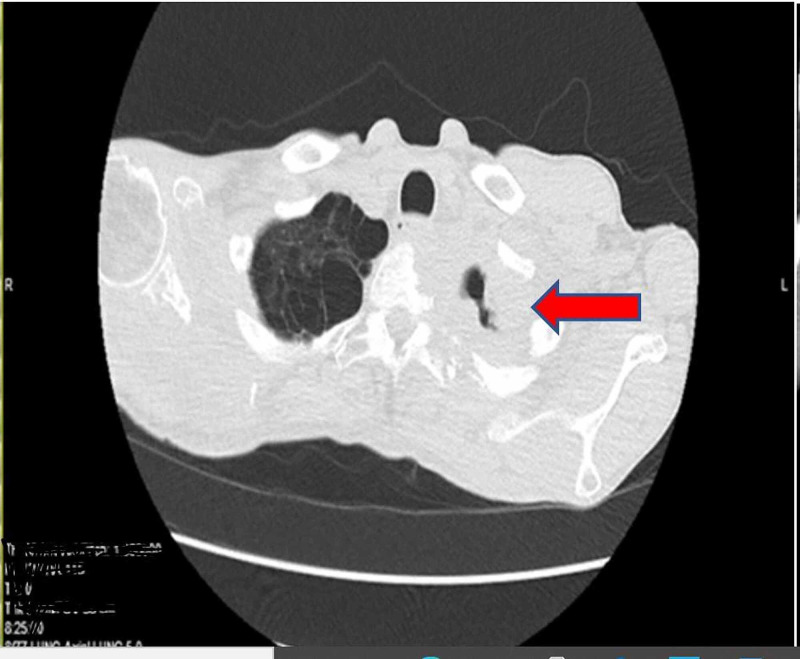
Axial section CT chest without contrast. Red arrow pointing to apical mass.

**Figure 3:**
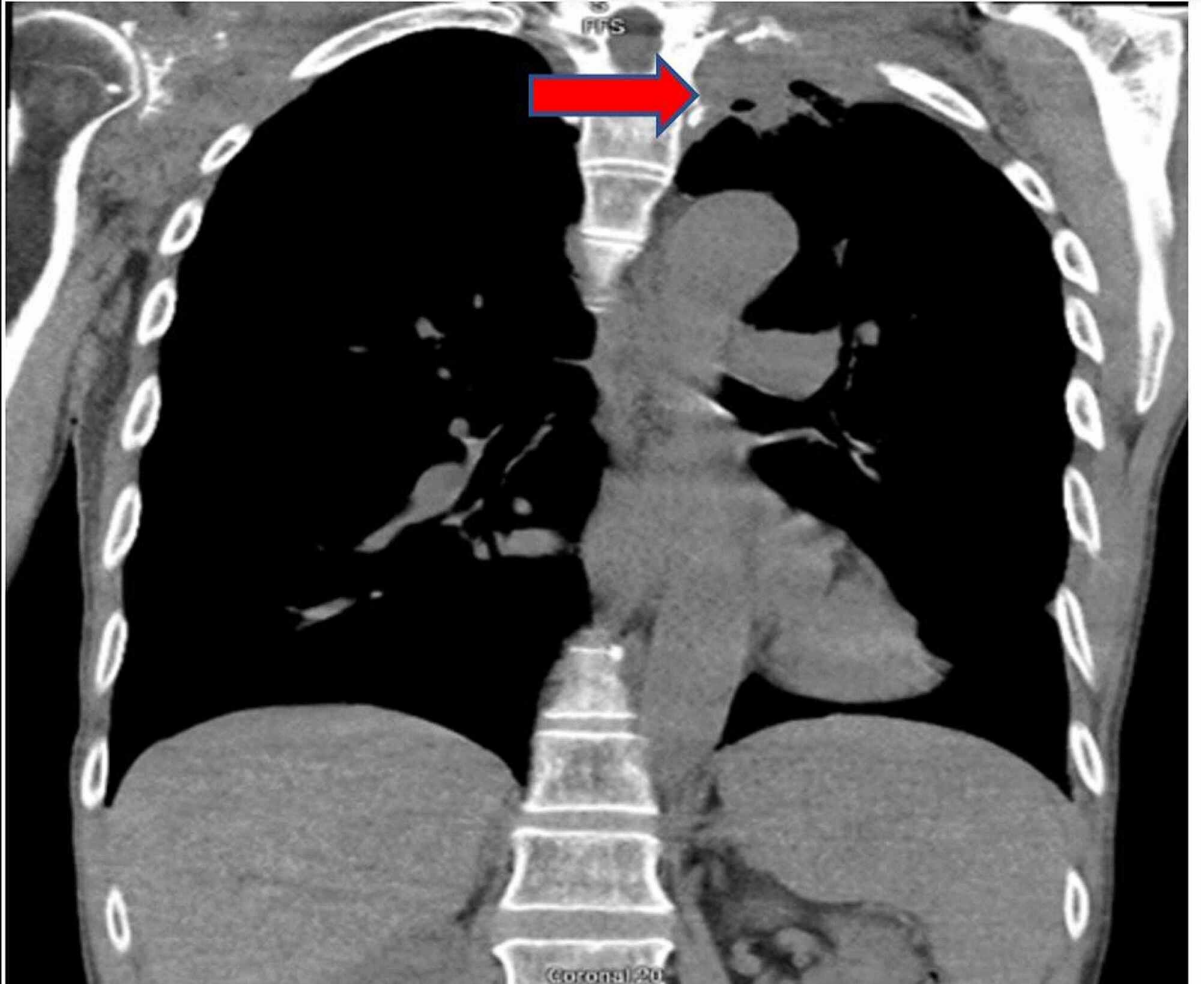
Coronal section CT chest. Red arrow showing mass on left side.

Laboratory results revealed anemia with a hemoglobin of 11.6 (normal value 13.5-18 g/dl), hematocrit of 37.4% (normal value 42-54%), ferritin of 472 ng/ml (normal value 23.9-336.2 mg/ml), normal serum iron of 65 µg/dl (normal value 50-212 µg/dl). The right shoulder radiography described a destructive lesion of the scapula with concern for malignancy, either primary or metastatic disease (Figure 1). Chest radiography was a supine film of poor quality and did not demonstrate any abnormality. Given his diffuse back pain complaint, he had CT of the cervical, thoracic, and lumbar spine, which revealed metastatic disease in the thoracic spine and a left apical mass. CT chest identified a soft tissue mass at the left lung apex with extension into the pleural surface, associated osseous destructive changes of the adjacent ribs, and extensive osseous destructive changes involving the right scapula (Figures 2, 3).

**Figure 4:**
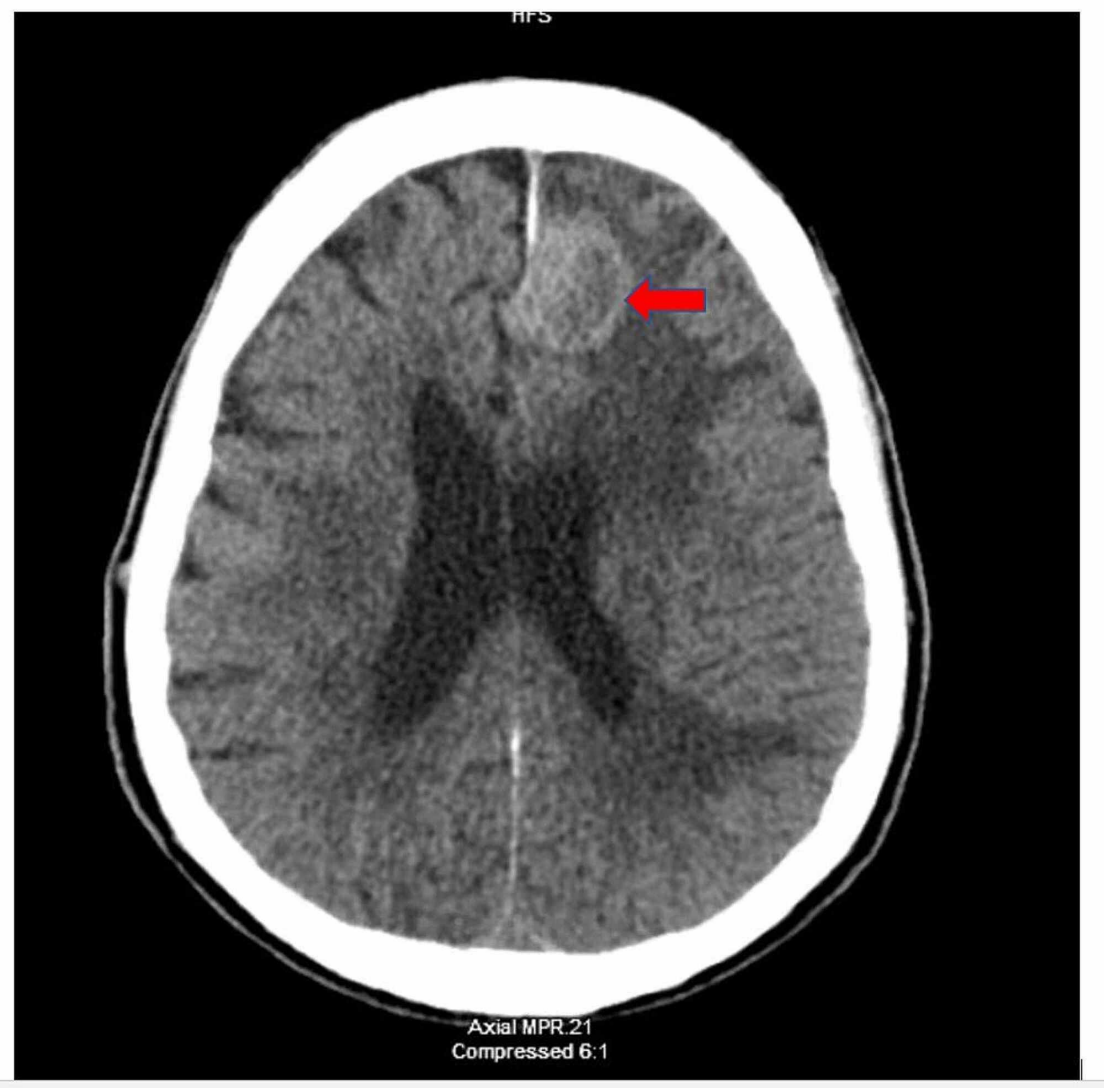
CT brain without contrast, axial section. Red arrow showing frontal lesion with surrounding edema.

**Figure 5:**
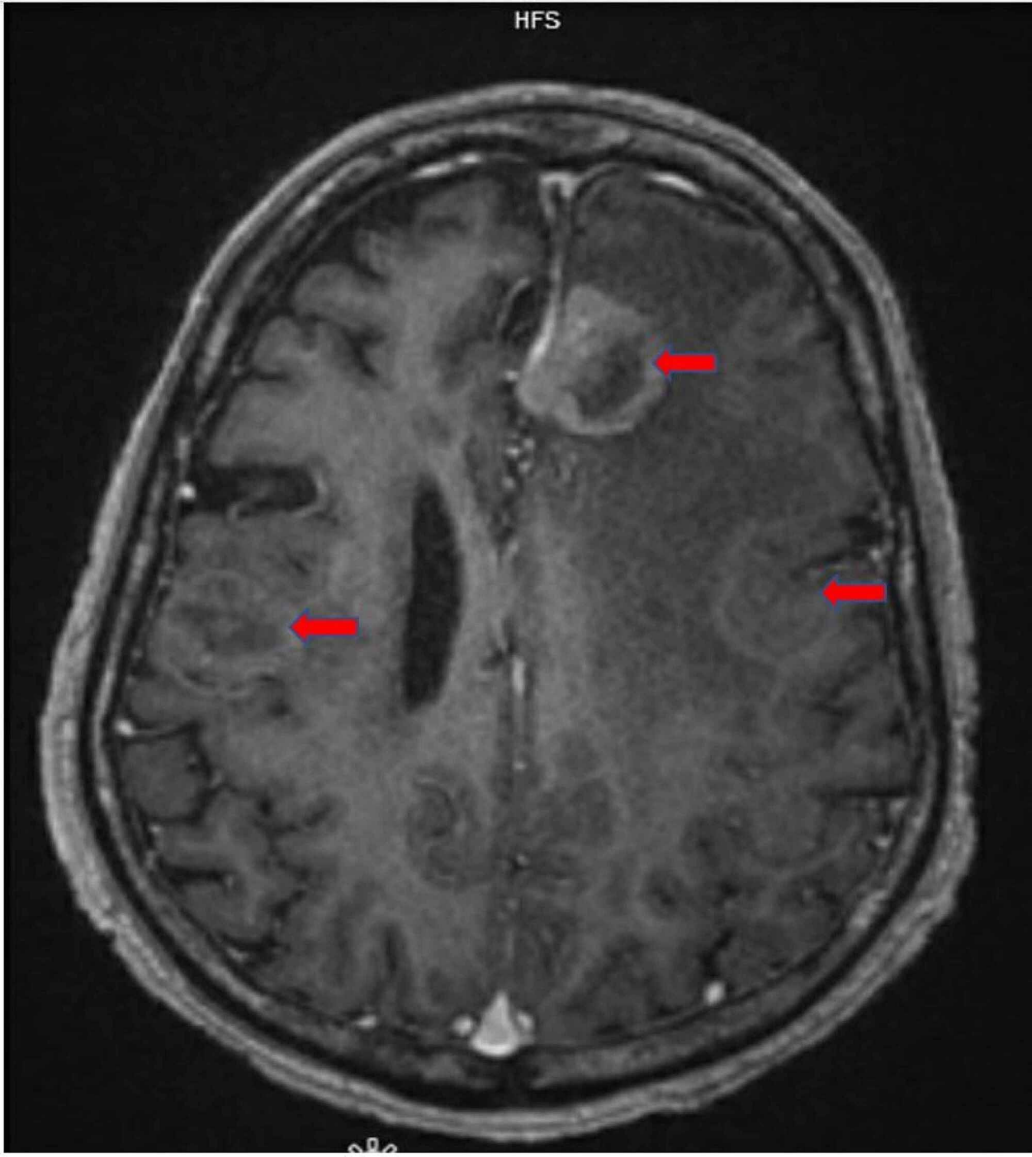
MRI brain with contrast, axial section. Red arrow showing multiple lesions with surrounding edema.

**Figure 6:**
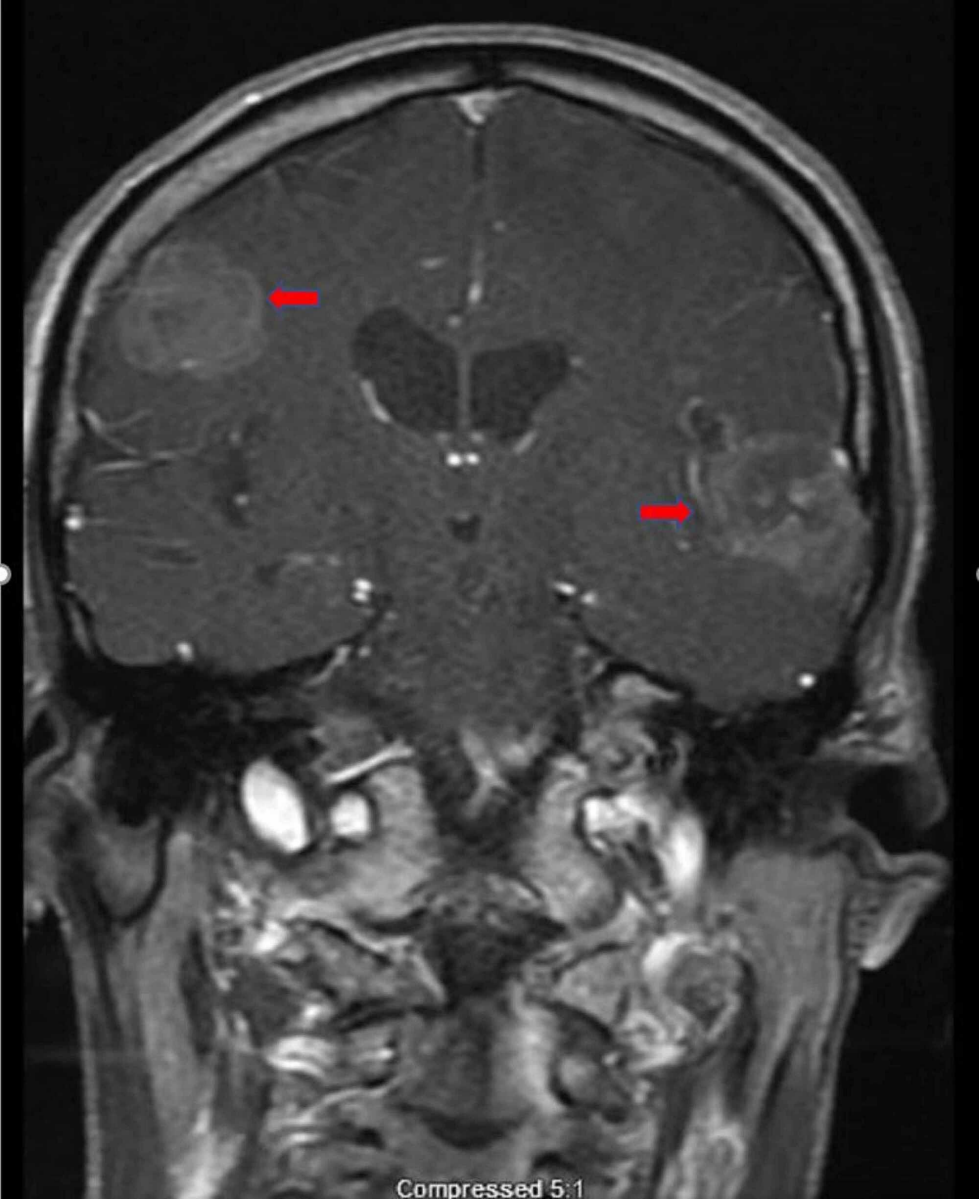
MRI brain with contrast, coronal section. Red arrows showing metastatic lesions.

**Figure 7:**
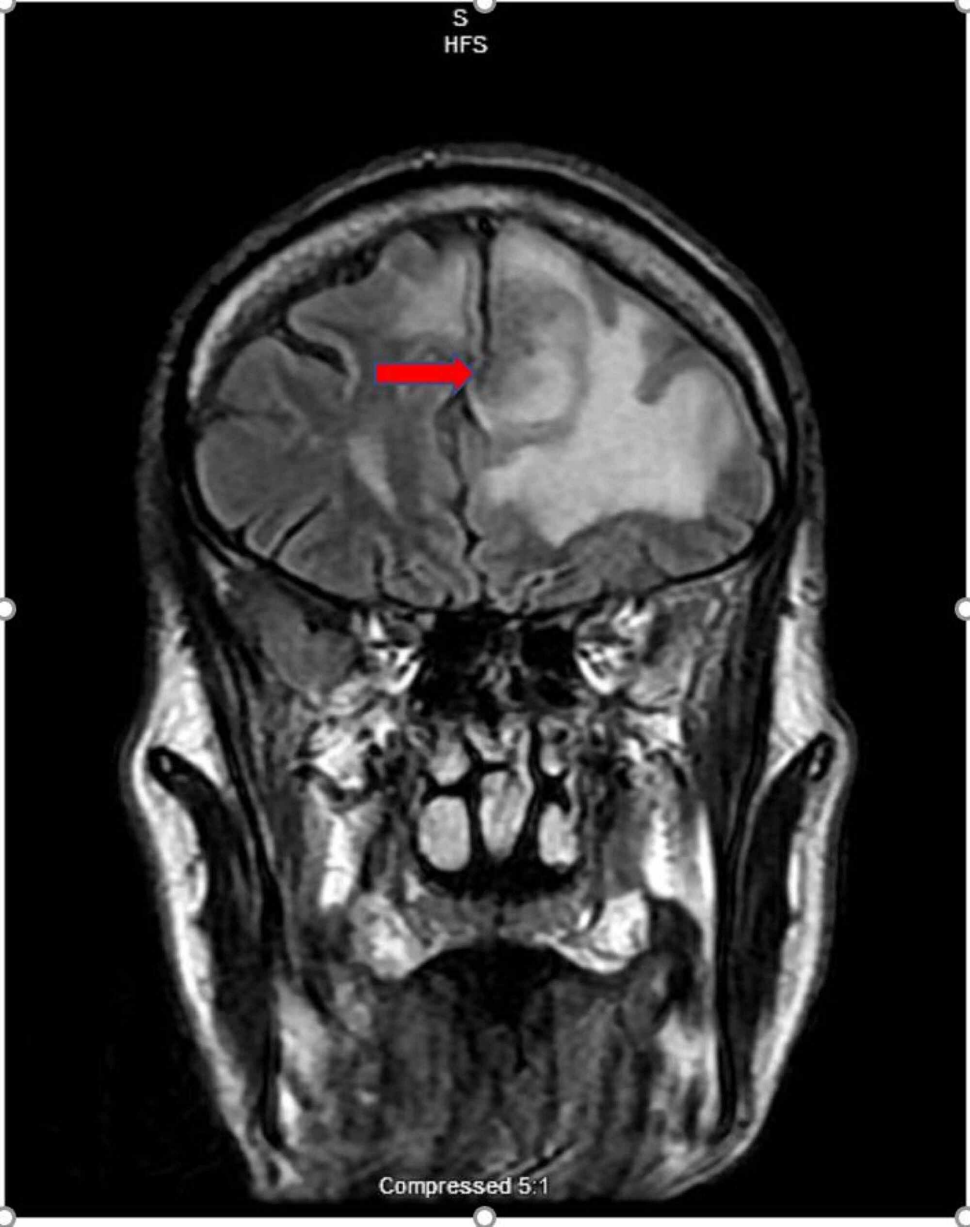
MRI brain, coronal flair section. Red arrow showing frontal lesion with surrounding edema.

CT brain revealed multiple intraparenchymal masses with surrounding mass effect and vasogenic edema concerning metastatic disease (Figure 4). MRI brain also revealed multiple enhancing brain lesions with pronounced surrounding vasogenic edema in the left frontal, left temporal, right frontal, right frontoparietal, and right posterior parietal (Figures 5, 6, 7).

 

**Figure 8:**
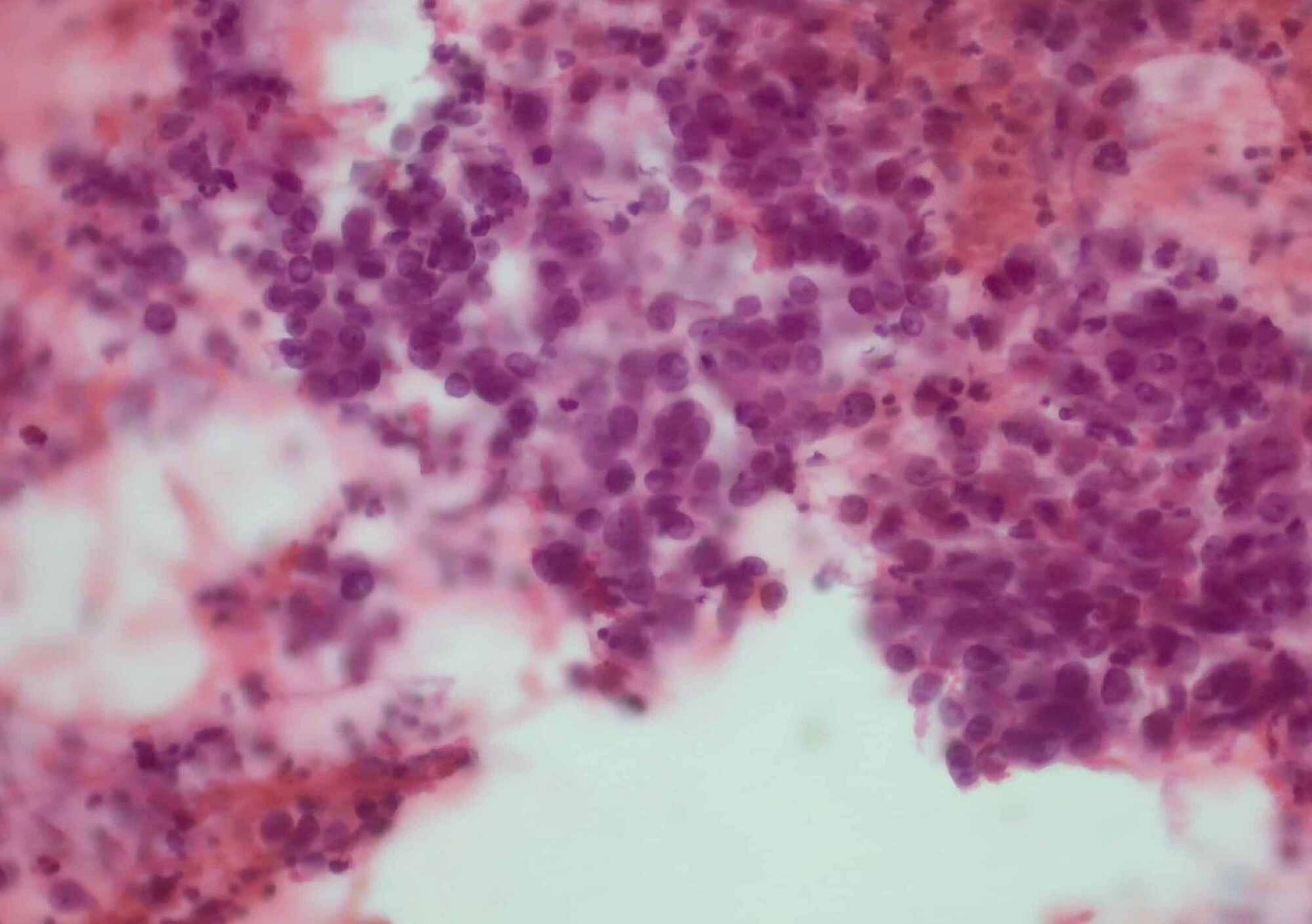
FNA slide with H&E stain showing malignant cells in sheets in a background of acute inflammation (x200 magnification). FNA: fine-needle aspiration; H&E: hematoxylin and eosin

**Figure 9:**
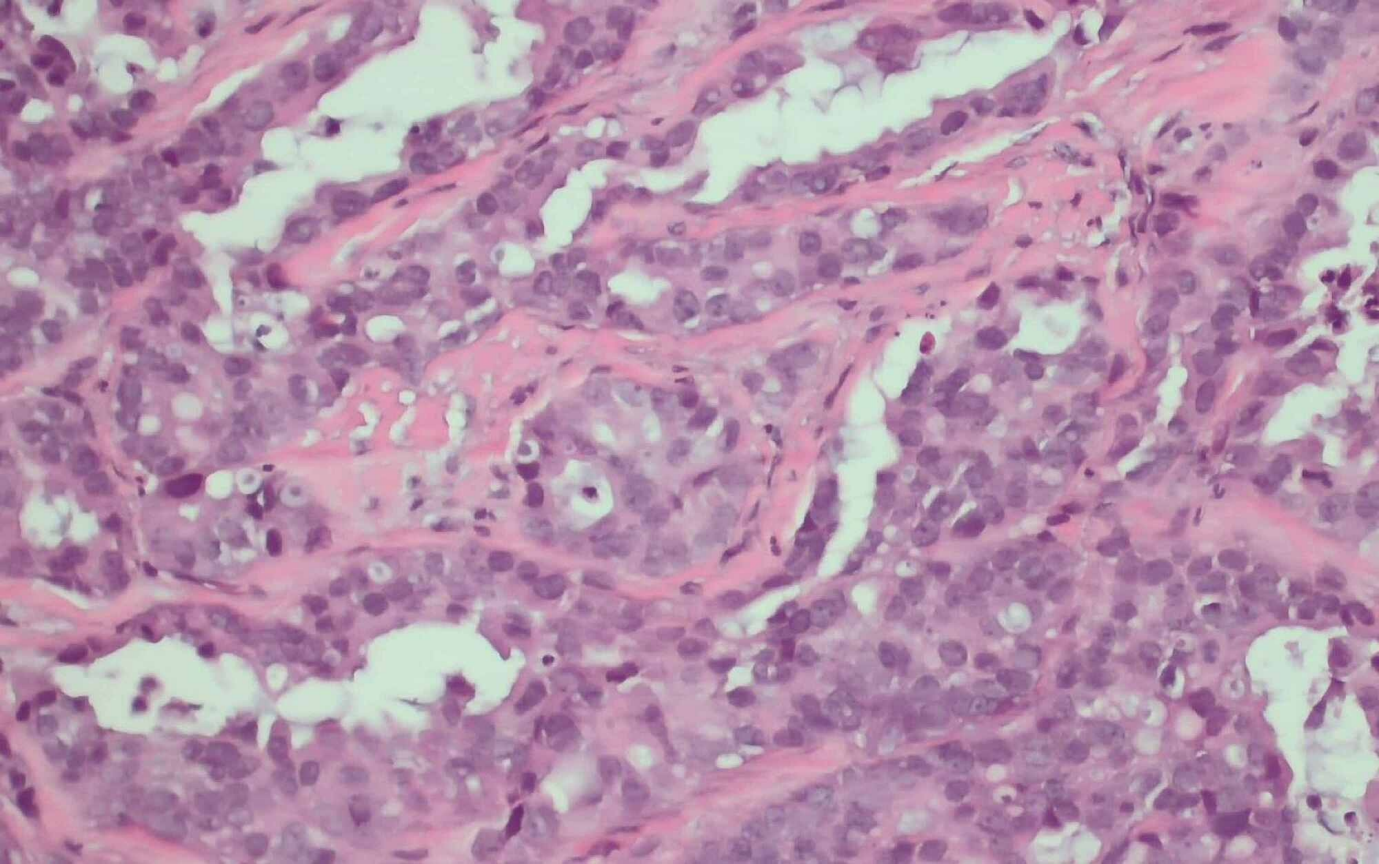
Core biopsy with H&E stain showing malignant glandular elements consistent with adenocarcinoma (x200 magnification). H&E: hematoxylin and eosin

**Figure 10:**
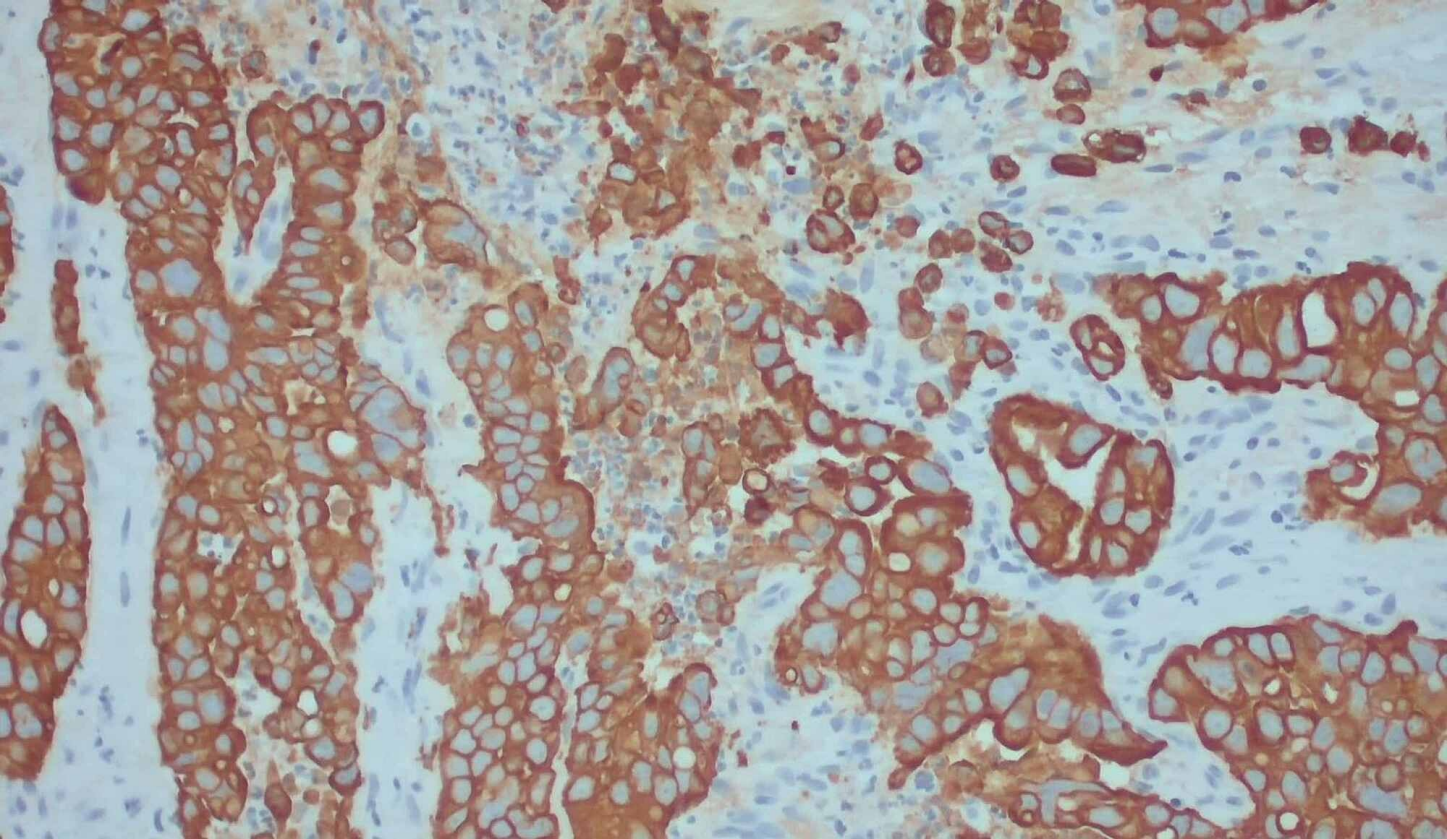
With CK7 immunostain on the core biopsy showing positivity in the tumor (x200 magnification). CK7: cytokeratin 7

**Figure 11:**
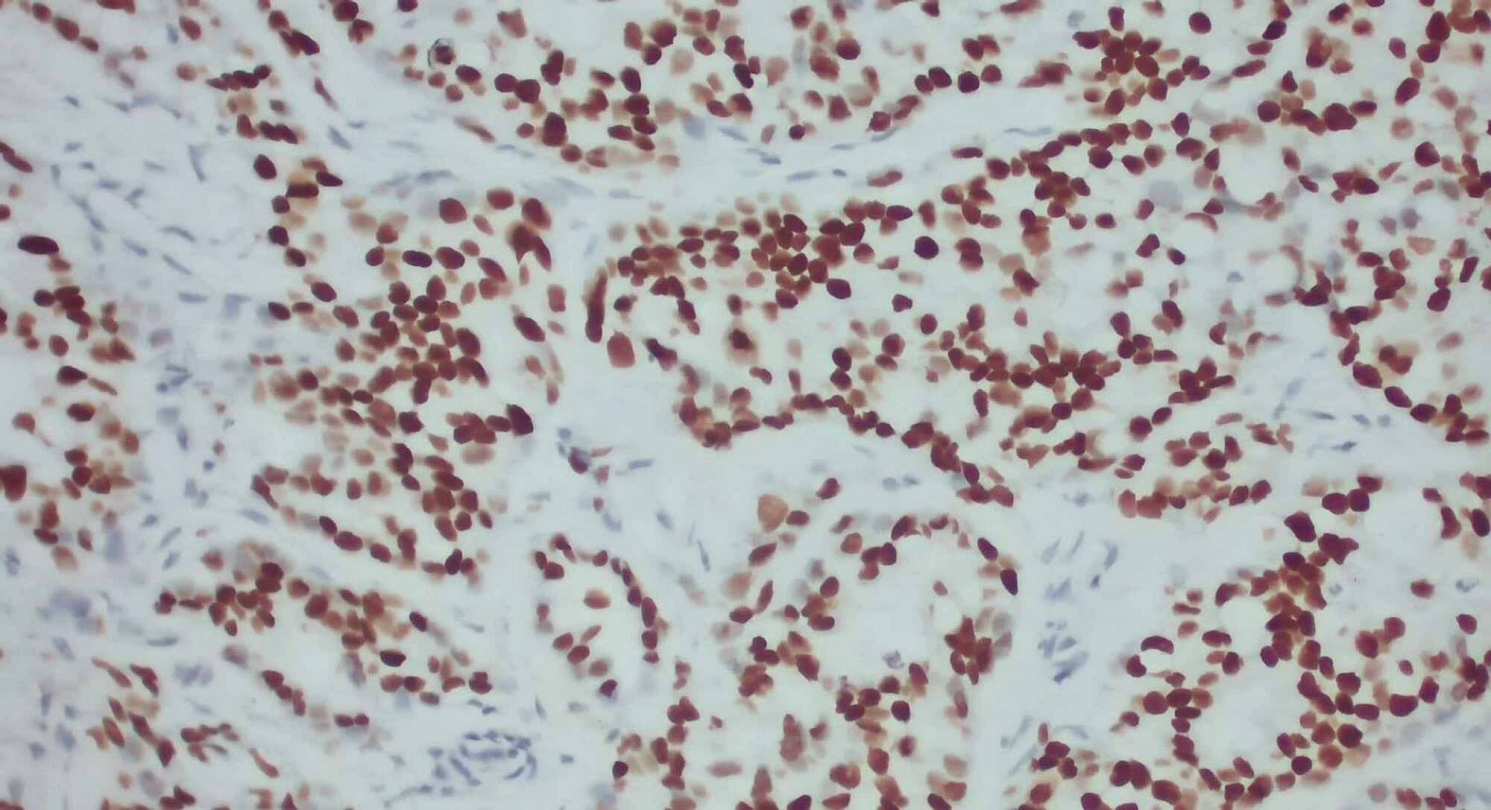
With TTF1 immunostain showing strong nuclear positivity (x200 magnification). TTF1: thyroid transcription factor 1

To accurately assess the etiology of the tumors, a biopsy of the supraclavicular mass was done. The biopsy depicted lung tissue with adenocarcinoma with negative Kirsten rat sarcoma viral oncogene homolog (K-Ras), epidermal growth factor receptor (EGFR), B-raf proto-oncogene (BRAF), C-ros oncogene 1 (ROS1), and anaplastic lymphoma kinase (ALK) rearrangement. The phenotype cytokeratin 7 (CK 7) positive/thyroid transcription factor 1 (TTF1) positive is consistent with adenocarcinoma of lung primary. Images from the biopsy with histochemical stains are given in Figures 8, 9, 10, 11.

The patient was treated with intravenous steroids for cerebral edema and levetiracetam for seizure prophylaxis. He also received radiation therapy to the spine and right shoulder for pain control.

Given the extent of metastasis and the feeble performance status, the overall prognosis was poor; the patient declined chemotherapy and was discharged to a rehabilitation facility with plans to follow-up with oncology. 

## Discussion

In the 1900s, Pancoast described three cases of superior pulmonary sulcus tumor as tumor 'arising from the fifth brachial cleft's epithelial rests’ [2]. This was later revisited in 1932 by Tobias, who described the tumor site of origin as the bronchopulmonary tissue, hence the syndrome's name as Pancoast-Tobias syndrome [3].

Pancoast tumors constitute 3-5% of all lung cancers [4]. Historically, the majority of Pancoast tumors reported were a squamous cell in origin; however, there is an increasing number of cases of adenocarcinoma [2,4-6]. This may be explained by an overall rise in adenocarcinoma cases and a decline in squamous cell carcinoma. It has been hypothesized that the transition from unfiltered cigarettes to low-tar filter cigarettes has resulted in more release of nitrosamines resulting in inhalation of tobacco-specific carcinogens distally toward the bronchoalveolar junction where adenocarcinomas often arise. Another proposed theory for increasing rates of adenocarcinoma is the increase in pollution from urbanization and increasing nitric oxide levels [7]. 

Risk factors associated with the development of Pancoast tumors are the same as those for any lung cancer, with cigarette smoking as the most prevalent etiology. Secondary smoke exposure, prolonged exposure to asbestos, and exposure to industrial elements are other contributing factors [8]. The average age at presentation is the sixth decade of life, with men affected more frequently than women [4,8]. Our patient's presentation was consistent with these observations. 

Pancoast-Tobias syndrome presents with a characteristic constellation of symptoms, including shoulder/arm pain, Horner's syndrome, and hand muscles' atrophy [4]. The symptoms develop secondary to the brachial plexus and superior sympathetic chain invasion. Shoulder pain is the presenting symptom in 96% of patients. This could be due to the regional spread of tumor in the shoulder joint or invasion of brachial plexus, both reported in the case of our patient who initially attributed the shoulder and the back pain to a recent fall.

The prevalence of Horner's syndrome has ranged from 14-50% in different case series [4]. Forty percent of patients with Pancoast tumors have symptoms of Horner's syndrome [8]. Horner's syndrome is described classically as a triad of ipsilateral ptosis, miosis, and anhidrosis. It is caused by the paravertebral sympathetic chain's invasion and the inferior cervical (stellate) ganglion by the tumor, hence the alternate name, oculosympathetic paresis. It was first described in 1869 by the Swiss ophthalmologist Johann Friedrich Horner, hence the name.

Horner's syndrome is detectable with a detailed physical examination as a difference in pupillary size or anisocoria being most noticeable with a droopy eyelid. Due to adequate constriction in bright light, one can easily miss subtleties in the miotic eye. The physiology behind it is that the iris constrictor muscle's unopposed parasympathetic action produces a smaller ipsilateral pupil. This is associated with a dilation lag or inability to dilate in darkness due to sympathetic fibers involvement. Pupillary inability to dilate can be tested by illuminating the patient's eyes tangentially from below with a hand-held flashlight and then abruptly turning the room lights out. The normal pupil will immediately dilate, but the Horner pupil begins to dilate several seconds later. Ptosis could be challenging to be appreciated during quick exams as it may be considered negligible to an untrained eye. It is explained by the paresis of the Müller muscle, a sympathetically innervated smooth muscle. Both eye findings were identified at our patient's physical examination. 

Anhidrosis is the most challenging to identify. Patients may not notice a difference in sweating in the face. This is explained by the fact that in the current times most places are temperature controlled. Our patient did not notice a difference in sweating between the two sides of the face. Although challenging, careful examination of the eyes and a high clinical index of suspicion is necessary to diagnose Horner's syndrome timely. The diagnosis of Pancoast syndrome is clinical and prompts further imaging required to delineate the extent of the disease and the structures involved [8].

Tumor extension into surrounding nerve roots, especially ulnar nerve roots (C8 and T1), occurs in approximately 8-22% of Pancoast tumor and may result in weakness and atrophy of the intrinsic muscles of the hand, paresthesia over the distribution of 4th and 5th digits of hand and medial aspect of arm and forearm. In some cases, the patient may exhibit a loss of the triceps reflex [6]. Burning pain in the axilla and abnormal sensation in the intercostobrachial nerve territory have also been reported in the literature [9].

Management varies depending on the extent of the disease and functional status. Superior sulcus tumors, by definition, are T3 lesions due to involvement of the chest wall. T4 lesions would indicate the invasion of the brachial plexus, mediastinal structures, or vertebral bodies, as in our patient's case. 

The prior practice was a bimodal approach to treatment with radiation therapy and surgical resection. In the 1990's the overall five-year survival rate after combined preoperative radiotherapy and extended surgical resection was generally around 20-35% [4]. The trimodal approach is currently a standard of treatment with preoperative chemotherapy with platinum-based regimens/induction chemotherapy and external beam radiotherapy followed by surgery [10]. A recent review article compared radiotherapy followed by surgery vs. trimodal approach (induction chemotherapy, radiotherapy, and surgery) and revealed a two-year survival rate in the radiation therapy (RT) group from 22-49% vs. 70-93% in the trimodal therapy group. Most patients had T3 disease, unlike our patient [11].

Extensive extra-thoracic metastatic disease and positive mediastinal nodes are a contraindication to surgery, and in such cases, palliative therapy is preferred [8]. The presence of tumor markers like EGFR and KRAS may have some utility due to the availability of biologic antineoplastic agents like EGFR tyrosine kinase inhibitors and carries a good prognostic due to the use of targeted therapy based on the presence of specific mutations. Another aspect is that targeted therapy may enable tumor shrinkage to resectable size. However, overall clinical utility is unclear currently [12].

Clinical factors associated with improved survival include good performance status, localized disease, a weight loss of less than 5% of total body weight, and achievement of local control and pain relief after the treatment [2]. Our patient had a primary apical tumor with supraclavicular lymph node involvement and metastasis to the brain and bones, with an overall poor prognosis due to decreased functional status (Karnofsky performance status score of 40 points), severe protein-calorie malnutrition, and extensive metastatic disease. The palliative treatment, which our patient opted for, includes radiotherapy to the brain and spine for pain control, dexamethasone to reduce surrounding brain edema associated with metastatic lesions, and levetiracetam for seizure prophylaxis.

## Conclusions

This case report highlights the importance of careful assessment of patients presenting with Horner's syndrome. Expediting the diagnosis is crucial and may influence survival in patients with lung adenocarcinoma.

## References

[1] Setzer M, Robinson LA, Vrionis FD (2014). Management of locally advanced pancoast (superior sulcus) tumors with spine involvement. Cancer Control.

[2] Arcasoy SM, Jett JR (1997). Superior pulmonary sulcus tumors and Pancoast’s Syndrome. N Engl J Med.

[3] Panagopoulos N, Leivaditis V, Koletsis E (2014). Pancoast tumors: characteristics and preoperative assessment. J Thorac Dis.

[4] (2020). Pancoast Tumor. https://litfl.com/pancoast-tumour.

[5] Hilaris BS, Martini N, Wong GY, Nori D (1987). Treatment of superior sulcus tumor (Pancoast tumor). Surg Clin North Am.

[6] Gundepalli SG, Tadi P (2020). Cancer, lung Pancoast (superior sulcus Tumor). [Updated August 10, 2020]. StatPearls.

[7] Chen F, Jackson H, Bina WF (2009). Lung adenocarcinoma incidence rates and their relation to motor vehicle density. Cancer Epidemiol Biomarkers Prev.

[8] Karl J D'Silva, MD; Chief Editor: Nagla Abdel Karim, MD MD, PhD PhD (2020). Pancoast Syndrome. Updated: Jan 21.

[9] Kanagalingam S, Miller N (2015). Horner syndrome: clinical perspectives. Eye Brain.

[10] Foroulis CN, Zarogoulidis P, Darwiche K (2013). Superior sulcus (Pancoast) tumors: current evidence on diagnosis and radical treatment. J Thorac Dis.

[11] Buderi SI, Shackcloth M, Woolley S (2016). Does induction chemoradiotherapy increase survival in patients with Pancoast tumour?. Interact Cardiovasc Thorac Surg.

[12] Sun L, Zhang Q, Luan H, Zhan Z, Wang C, Sun B (2011). Comparison of KRAS and EGFR gene status between primary non-small cell lung cancer and local lymph node metastases: implications for clinical practice. J Exp Clin Cancer Res.

